# Building a Social Network One Choice at a Time

**DOI:** 10.1371/journal.pone.0133463

**Published:** 2015-07-17

**Authors:** Jordan W. Suchow

**Affiliations:** Department of Psychology, Harvard University, Cambridge, Massachusetts, 02138, United States of America; CSIC-Univ Miguel Hernandez, SPAIN

## Abstract

Newcomers to a social network show *preferential attachment*, a tendency to befriend those with many friends. Here, we show that preferential attachment is equivalent to a form of ‘probability matching’ commonly found in studies of decision-making. This equivalence, whereby newcomers probability match to a social signal akin to popularity, marries network science to the study of decision-making and raises new questions about how individual psychology impacts the social structure of groups. We asked people to view a visualization of a social network and to select group members whom they would like to meet and befriend. People varied in how strongly they weighed popularity and this was mildly correlated with aspects of their personality. Individual differences in preferential attachment affect the structure and connectivity of the network that emerges.

## Introduction

Many networks grow one by one as newcomers join an existing infrastructure. The World Wide Web grows when a new page links to an established site, the citation network of the scientific literature grows when a new article cites a published paper, and a social network grows when someone makes a new friend. In each case, the newcomer links up with a chosen subset of the network’s current members, and this choice is known to affect the global structure of the network that emerges [1–3]. For example, if newcomers to a social network tend to befriend those who already have many friends, a principal known as preferential attachment, the resulting network will have a hierarchy of hubs and outsiders, characteristic of many real-world networks [1].

Preferential attachment can be framed in terms of decision theory. One well-known principal of decision-making, Luce’s choice axiom, stipulates that when faced with a choice among alternatives, a decision maker will exhibit ‘matching behavior’, selecting options with probability proportional to their value [4, 5]. Matching behavior was originally studied in the context of learning theory, where value is defined as the expected reward; thus if two levers offer reward in a ratio of 2:1, an individual who displays matching behavior will press the more rewarding lever twice as often [6, 7]. Here, in the context of network construction, value is assumed to be social and akin to popularity. Specifically, we define the value attributed to the choice of befriending a certain group member as the number of connections between that member and all the others. Sensitivity to popularity can arise as a byproduct of other mechanisms, such as imitation [8] or *homophily*, a tendency to associate with others who are similar [9, 10].

In practice, it is common to consider a generalization of matching behavior in which a real-valued parameter *L* determines the decision maker’s sensitivity to the value [7, 11]. In the softmax generalization of matching behavior, the probability of selecting option *a* from the set of alternatives *A* is given by

$$P\left(a\right)=\;\frac{v{\left(a\right)}^{L}}{\sum_{b\in A}v{\left(b\right)}^{L}},$$ (1)

where *v*(*x*) is the value generated by *x* and where *L* determines the decision maker’s sensitivity to the value [7, 11]. If *L* is zero, the newcomer is blind to value and therefore disregards popularity. If *L* is positive, the newcomer is sensitive to value and prefers to befriend those who are popular. If *L* is negative, the newcomer is sensitive to value but behaves in the opposite manner, tending to befriend those who are unpopular.

**Fig 1:**
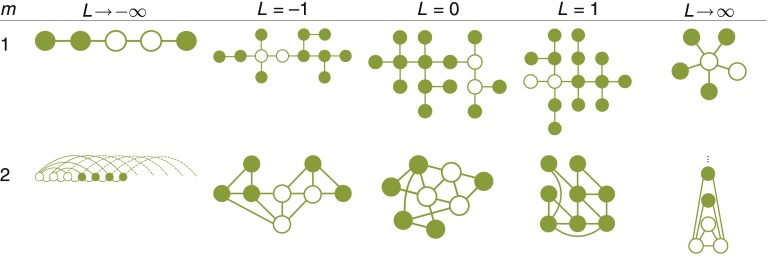
Social networks that arise when newcomers use popularity to guide their selection of friends. Starting with Euler in 1736, the study of network topology has been couched in terms of graph theory, which represents individuals as nodes (drawn here as green circles) and a connection between two individuals as an edge between two nodes (green lines). Together, the nodes and edges define a graph. The arrangement of the nodes on the page is nonessential; what matters is who links to whom. The networks shown here were constructed through a process in which the newcomer samples *m* times (without replacement) from the distribution defined by Eq 1 and links up with whomever is selected [1]. Each network began as a complete (i.e., fully-connected) graph with *m*+1 nodes (open circles) and grew with the arrival of newcomers (filled circles).

The value of *L* used by a newcomer determines the structure of the resulting social network (Fig 1). For example, setting *L* = 1 produces a scale-free network [1], setting *L* → ∞ produces a star [3], and setting *L* → –∞ produces a new family of networks, the demophobics (Fig 1, leftmost column).

By framing preferential attachment in terms of decision-making, it opens the door to new questions about social networks that are inspired by the psychology of individual decision-making. As a first step through that door, we relax the typical assumption in network science that the whole population shares a common value of *L*, and instead suppose that, like many psychological traits, it varies from person to person. We then measure these individual differences and determine whether they are correlated with aspects of the decision-maker’s personality.

## Methods

### Participants

We recruited 600 people using Amazon Mechanical Turk, an online labor market where participants complete brief tasks for pay [12, 13]. Recruitment was limited to participants from the United States of America. Demographic studies of Mechanical Turk participants have found that such workers are fairly representative of the population of US internet users, though on average they are younger, have lower income, are more educated, and include more females [14–16].

### Ethics

Experiments were performed in accordance with Harvard University regulations and were approved by the Committee on the Use of Human Subjects in Research under the IRB for the Faculty of Arts and Sciences.

### Procedure

**Fig 2:**
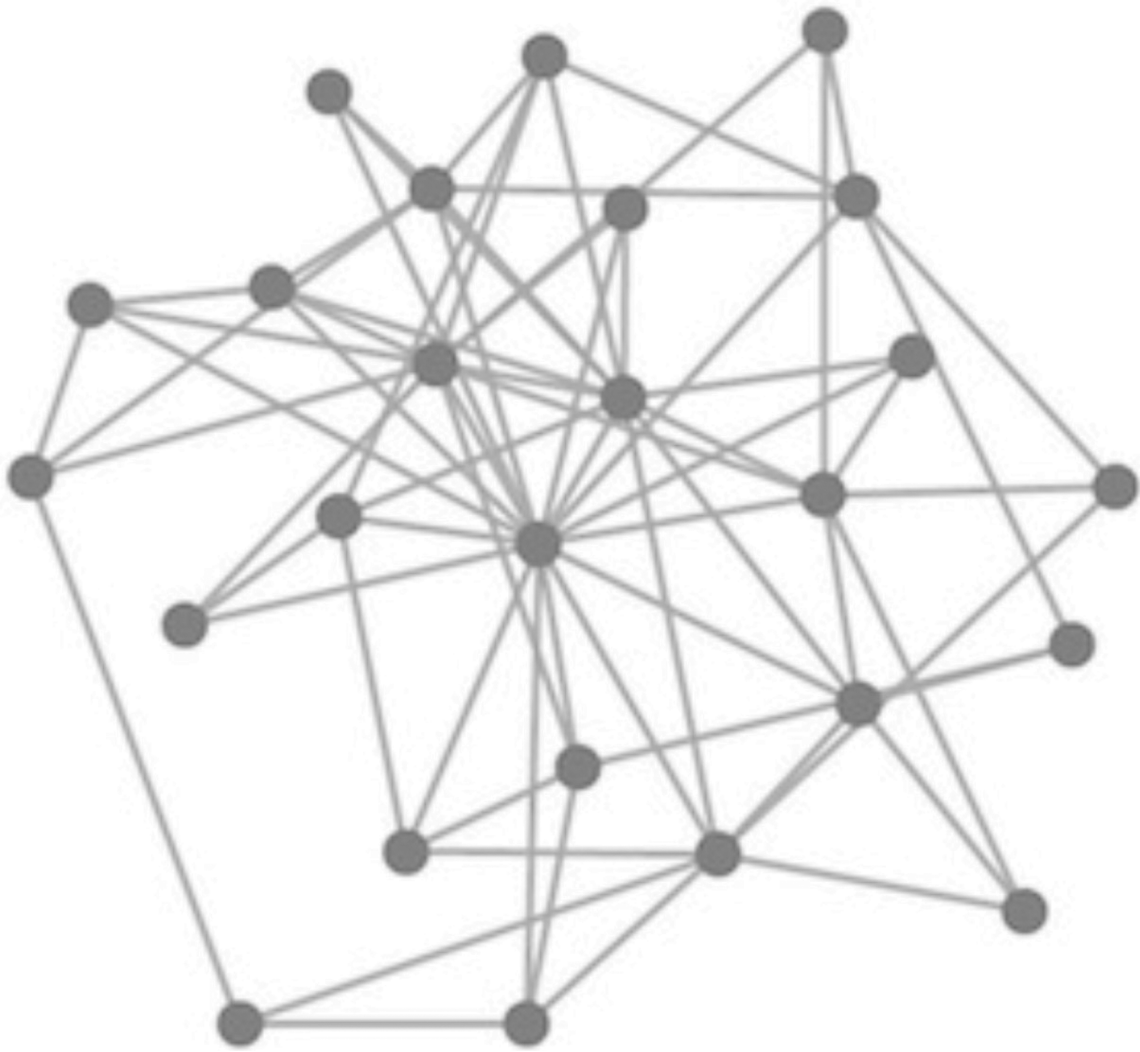
A visualization of a social network, used as the stimulus in the experiment. The image appeared to the participants exactly as it appears to you, the reader.

All participants were provided with the same image (seen in Fig 2), which was said to depict the structure of a social network (i.e., who is friends with whom). After looking at Fig 2 for at least 15 seconds, participants selected two nodes from the network, representing two people whom they would like to meet and befriend. Afterwards, for each selected node, participants reported the number of links that they perceived between that node and the others in the network. Finally, participants completed a 10-item version of the Big Five Inventory, a test that assesses personality along five dimensions: openness, conscientiousness, extraversion, agreeableness, and neuroticism [17]. The time required by the participants to complete all tasks, including reading the instructions, was 127 s (95% CI [120, 135], bootstrapped with 10^6^ samples, as per all CI’s reported here).

### Stimuli

The stimulus was a visualization of a social network (Fig 2), created through the following procedure. First, we used the Barabási–Albert model to construct a 25-node scale-free network [1]. This is equivalent to the model based on probability matching, outlined in the caption of Fig 1, with parameters *L* = 1 and *m* = 3. Next, we drew the network as a force-directed graph, arranged using the D3 library [18]. Force-directed graphing is a visualization technique that assigns attractive forces between nearby nodes and repulsive forces between distant nodes, in such a way that edges have similar lengths and rarely cross [19]. Nodes were drawn as dark grey circles and edges were drawn as thin lines connecting the nodes. Visualizing the network as a force-directed graph trades off between two conflicting desires: first, to communicate the network's structure in a format that is easily digested, and second, to avoid biasing the participant's choice by leading them to make a decision based on an incidental property of the visualization and not the network's structure. In force-directed graphing, the structure of the network determines the placement of the nodes in the image, with more central nodes placed more centrally. Choosing a format of visualization that is guaranteed to be inert with respect to the participant's choice requires a deeper understanding of people's internal representation of network structure.

### Detecting individual differences in *L*

To test for the presence of individual differences in preferential attachment, we first measured the correlation between the value of *L* used for each participant’s first and second choice of friends. The best-fit value of *L* was computed separately for each participant, and separately for the first and second choice. Eq 1 gives the probability density function of the participant's decision process. The first node was chosen from all the nodes in the network shown in Fig 2. The second node was chosen from the same set of nodes, but with the participant's first choice excluded. We placed a truncated-gaussian prior over *L* (*μ* = 0, s.d. = 5, truncated at ±10). The maximum a posteriori value of *L* was inferred from the experimental data using the Metropolis–Hastings variant of Markov Chain Monte Carlo (MCMC), which provides a sampling-based approximation to a full posterior distribution [20]. MCMC was implemented using a modified version of the MemToolbox [21], which, alongside the raw data (S1 Dataset) and analysis scripts (S1 Scripts), is available in Supporting Information. The MCMC procedure used five chains that were started at *L* = –8, –2, 0, 2, and 8, with gaussian proposal steps (standard deviation 0.1), tuned every 200 steps. Convergence between the chains was detected using the method of Gelman and Rubin [22]. We collected 6,000 samples from the converged chains. Individual differences in *L* lead to a positive correlation between the first and second selections, with each participant using a consistent value *L*, but with that value of *L* differing across participants.

Next, as a more thorough test of individual differences, we compared two models—one without individual differences, and the other with them. In the first model, there is a shared (but unknown) value of *L* across all the participants. As with individual choice behavior, we placed a truncated gaussian prior over *L* (*μ* = 0, s.d. = 5, truncated at ±10). In the second model, *L* varies across participants according to a gaussian distribution with unknown mean and variance. The prior on the mean was the same as in the fixed-*L* model; the prior on the standard deviation was lognormal (*μ* = 0, s.d. = 1). Maximum a posteriori means and standard deviations, along with their credible intervals, were computed directly from these posterior samples. Finally, the two models were pit against each other using the Akaike Information Criterion with a correction for finite data [23].

## Results and Discussion

**Fig 3:**
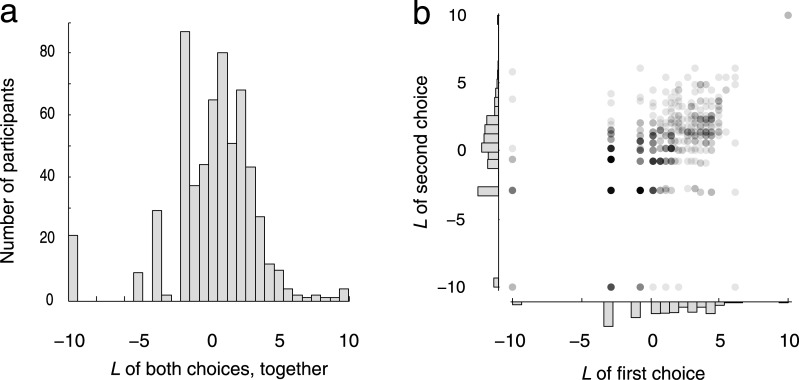
Individual differences in sensitivity to popularity when selecting whom to befriend. (A) Distribution of the best-fit value of *L* across participants. (B) Correlation between the best-fit values of *L* for a participant's first and second selections, fit separately.

After viewing a visualization of a social network, participants selected two people whom they would like to meet and befriend. The average value of *L* across the population was 0.68 (95% credible interval [0.35, 1.00]), with a standard deviation of 3.1 (95% credible interval [2.8, 3.5]; Fig 3a). With a population average above zero and below 1, on average participants fell in the range of sublinear preferential attachment [24–27], but with individual participants falling everywhere along the continuum from superlinear, linear, and sublinear preferential attachment, to insensitivity, to sublinear, linear, and superlinear antipreferential attachment. There was a positive correlation between the value of *L* used by a participant for the first and second selections (*r* = 0.49, 95% CI [0.41, 0.57]; Fig 3b), which is suggestive of individual differences in *L*.

The presence of these individual differences was confirmed by comparing the goodness of fit of two models—one without individual differences in *L*, and the other with such individual differences. The model with individual differences provided a better fit to the data (difference in Akaike information criterion, 283). Furthermore, an individual’s best-fit value of *L* across the two selections was correlated with the Big Five personality trait of extraversion (*r* = 0.17, 95% CI [0.09, 0.25]), but not with agreeableness (*r* = 0.03, 95% CI [–0.05, 0.10]), conscientiousness (*r* = –0.01, 95% CI [–0.09, 0.06]), neuroticism (*r* = –0.06, 95% CI [–0.14, 0.01]), or openness (*r* = –0.04, 95% CI [–0.11, 0.04]). The mild correlation with extraversion suggests that the participants used a decision process relevant to everyday social interaction, despite the task’s simple and abstract format. Extraversion, in particular, is linked to sensitivity to social reward signals [28].

**Fig 4:**
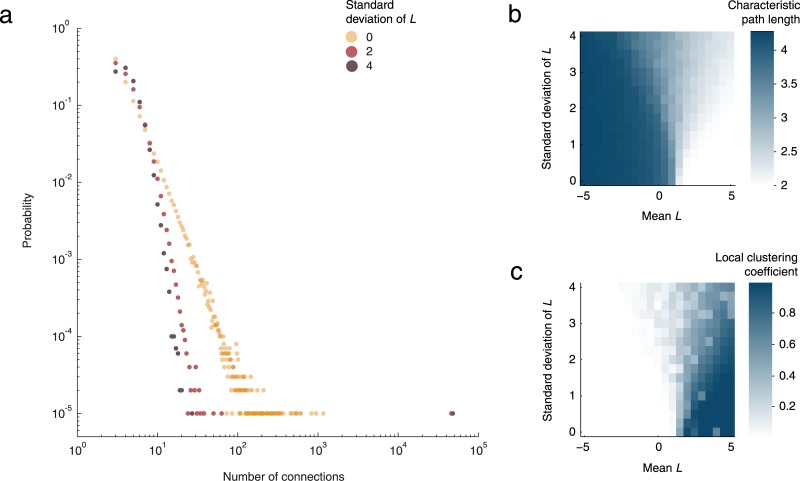
The structure and connectivity of a growing network depends on the policy used by a newcomer when selecting its connections. (a) The degree distribution specifies how likely it is for a person to have a particular number of connections. These are the degree distributions for three networks that vary only in the standard deviation of *L* across the population (10,000 individuals, *L* = 1, *m* = 3). Introducing individual differences bends the degree distribution away from being a straight line, the signature of a scale-free network. (b) Another measure of network connectivity that is affected by the variability is the characteristic path length—the average distance between individuals. Notice the interaction between the direction of the effect and the value of *L* with no effect of individual differences at *L* = 1. (c) A third affected measure of network connectivity is the local clustering coefficient, the proportion of possible connections among one’s friends that actually exist, averaged across all people. We tested mean values of *L* between –5 and 5 in steps of 0.25, and standard deviations of *L* between 1 and 4 in steps of 0.25. Each combination of parameter values was run once, with *m* = 3 and 10,000 nodes.

How do individual differences affect the structure and connectivity of the resulting networks? Simulations revealed an interaction between the average value of *L* across the population and the impact of individual differences on network connectivity (Fig 4). When *L* is less than 1, individual differences push the network further towards a small-world architecture, with short paths between members and high clustering, where one’s friends tend to know each other. When *L* is greater than 1, the opposite occurs: individual differences push the network away from being a small world. Critically, when *L* is close to 1, while the “scale-free” network topology is distorted (Fig 4a), the small-world architecture remains the same (Fig 4b and 4c). Thus, in our participants, whose average *L* is close to 1, the average behavior of the group tends to mitigate the impact of their individual differences, preserving the small-world architecture despite the variability.

In the same way that matching behavior can arise as a byproduct of other mechanisms, such as sampling-based approximations to rational inference [11, 29], preferential attachment can also arise as a byproduct of other mechanisms, such as imitation or homophily [8–10]. Framing preferential attachment in terms of the psychology of decision-making is a step towards a unified account of the construction of social networks, one in which the observed structure and connectivity of real-world networks provides a test bed for psychological theory, and in which psychological theory constrains the mechanisms that might give rise to that structure.

## Supporting Information

S1 Dataset Data collected in the main experiments. Contains the participants’ choices, the structure of the network they saw, and results of the personality survey.
(MAT)

S1 Scripts A directory of analysis scripts. (ZIP)

## References

[1] Barabási AL, Albert R (1999) Emergence of scaling in random networks. *Science*, 286(5439), 509–512. 10521342 10.1126/science.286.5439.509

[2] D'Souza RM, Krapivsky PL, Moore C (2007) The power of choice in growing trees. *The European Physical Journal B–Condensed Matter and Complex Systems*, 59(4), 535–543. 10.1140/epjb/e2007-00310-5

[3] Krapivsky PL, Redner S, Leyvraz F (2000) Connectivity of Growing Random Networks. *Physical Review Letters*, 85(21), 4629–4632. 11082613 10.1103/PhysRevLett.85.4629

[4] Herrnstein RJ (1961) Relative and absolute strength of response as a function of frequency of reinforcement. *Journal of the Experimental Analysis of Behavior*, 4, 267–272. 13713775 10.1901/jeab.1961.4-267 PMC1404074

[5] Luce RD (1959) *Individual Choice Behavior*: *A Theoretical Analysis*: New York: Wiley

[6] Estes WK (1957) Of models and men. *American Psychologist*, 12(10), 609–617. 10.1037/h0046778

[7] Sutton RS, Barto AG (1998) *Reinforcement Learning*: *An Introduction (Adaptive Computation and Machine Learning)*: The MIT Press.

[8] Ravi K, Prabhakar R, Sridhar R, Andrew T (1999) Extracting large-scale knowledge bases from the web. *Proceedings of the 25th International Conference on Very Large Data Bases*, 99, 639–650.

[9] McPherson M, Smith-Lovin L, Cook JM (2001) Birds of a feather: Homophily in social networks. *Annual Review of Sociology*, 27, 415–444.

[10] Papadopoulos F, Kitsak M, Serrano MA, Boguna M, Krioukov D (2012) Popularity versus similarity in growing networks. *Nature*, 489(7417), 537–540. 22972194 10.1038/nature11459

[11] Vul E (2010) *Sampling in human cognition*. PhD dissertation, Massachusetts Institute of Technology.

[12] Buhrmester M, Kwang T, Gosling SD (2011) Amazon's Mechanical Turk. *Perspectives on Psychological Science*, 6(1), 3–5. 10.1177/1745691610393980 26162106

[13] Mason W, Suri S (2012) Conducting behavioral research on Amazon’s Mechanical Turk. *Behavior Research Methods*, 44(1), 1–23. 10.3758/s13428-011-0124-6 21717266

[14] Berinsky AJ, Huber GA, Lenz GS (2012) Evaluating Online Labor Markets for Experimental Research: Amazon.com’s Mechanical Turk. *Political Analysis*, 20, 351–368.

[15] Ipeirotis P (2010) Demographics of Mechanical Turk. *CeDER working paper CeDER-10-01*, New York University, Stern School of Business.

[16] Ross J, Irani L, Silberman M, Zaldivar A, Tomlinson B (2010) Who are the crowdworkers? Shifting demographics in Mechanical Turk. *Proceedings of the 28th of the International Conference on Human Factors in Computing Systems*.

[17] Rammstedt B, John OP (2007) Measuring personality in one minute or less: A 10-item short version of the Big Five Inventory in English and German. *Journal of Research in Personality*, 41(1), 203–212. 10.1016/j.jrp.2006.02.001

[18] Bostock M, Ogievetsky V, Heer J (2011) D3 Data-Driven Documents. *IEEE Transactions on Visualization and Computer Graphics*, 17, 2301–2309. 22034350 10.1109/TVCG.2011.185

[19] Kobourov SG (2012) Spring embedders and force directed graph drawing algorithms. *arXiv*, arXiv:1201.3011.

[20] Andrieu C, De Freitas N, Doucet A, Jordan MI (2003) An introduction to MCMC for machine learning. *Machine Learning*, 50(1–2), 5–43.

[21] Suchow JW, Brady TF, Fougnie D, Alvarez GA (2013) Modeling visual working memory with the MemToolbox. Journal of Vision, 13(10), 9 10.1167/13.10.9 23962734 PMC4521709

[22] Gelman A, Rubin DB (1992) Inference from iterative simulation using multiple sequences. *Statistical Science*, 457–472.

[23] Akaike H (1974) A new look at the statistical model identification. *IEEE Trans*. *Automatic Control*, AC-19, 716–723.

[24] Newman ME (2001) Clustering and preferential attachment in growing networks. *Physical Review E*, 64(2), 025102. 10.1103/PhysRevE.64.025102 11497639

[25] Jeong H, Néda Z, Barabási AL (2003) Measuring preferential attachment in evolving networks. *Europhysics Letters*, 61(4), 567–572.

[26] Dereich S, Mörters P (2009) Random networks with sublinear preferential attachment: degree evolutions. Electronic Journal of Probability, 14(43), 1222–1267.

[27] Gabel A, Redner S (2013) Sublinear but never superlinear preferential attachment by local network growth. *Journal of Statistical Mechanics*: *Theory and Experiment*, 2013(02), P02043.

[28] Ashton MC, Lee K, Paunonen SV (2002) What is the central feature of extraversion? Social attention versus reward sensitivity. *Journal of Personality and Social Psychology*, 83(1), 245–252. 12088129

[29] Vul E, Goodman ND, Griffiths TL, Tenenbaum JB (2009) One and done? Optimal decisions from very few samples *Proceedings of the 31st Annual Conference of the Cognitive Science Society*. Amsterdam, Netherlands. 10.1111/cogs.12101 24467492

